# Modified Primary PSARP for an Adult Patient With Imperforate Anus With Rectoperineal Fistula in a Low‐Resource Setting: Case Report

**DOI:** 10.1155/cris/3272170

**Published:** 2026-09-28

**Authors:** Awol Yemane Legesse, Hale Teka, Mekonen Hafte, Sia Nyandemo, Sherif Emil

**Affiliations:** ^1^ Aberdeen Women’s Centre, Freedom Fistula Foundation, Freetown, Sierra Leone; ^2^ Department of Obstetrics and Gynecology, School of Medicine, Mekelle University, Mekelle, Ethiopia, mu.edu.et; ^3^ Maternal Health Africa, Freetown, Sierra Leone; ^4^ Department of Pediatric Surgery, McGill University Faculty of Medicine and Health Sciences, Montreal, Canada, mcgill.ca; ^5^ Mercy Ships, Freetown, Sierra Leone

**Keywords:** adult, case report, imperforate anus, PSARP

## Abstract

Anorectal malformations (ARMs) are congenital anomalies that include a variety of anomalies. An imperforate anus is the most typical presentation of all the ARMs. It can present with a fistula of the vestibule, vagina, fourchette, and perineum. Though in developed countries, it is diagnosed early and managed during the first few months of life, in low‐resource settings, patients present with ARM later in life. Our case presented a 24‐year‐old with an imperforate anus and rectoperineal fistula. This is the oldest patient to be reported in the literature with an imperforate anus. Modified posterior sagittal anorectoplasty was done. The technique employed is perineum‐preserving anal transposition. The procedure was successful. During 6 months of follow‐up, the reconstruction appeared smooth; she could control flatus and stool. She can differentiate between gas and stool. No constipation and no soiling. In low‐resource settings, patients present late during adulthood. Modified (perineum‐sparing) posterior sagittal anorectoplasty with anal transposition is an appropriate option for adult patients with ARMs.

## 1. Introduction

Anorectal malformations (ARMs) encompass a wide range of congenital anomalies of the lower segment of the gastrointestinal tract. They occur due to disruptions in embryogenesis caused by abnormalities in the formation, migration, or fusion of the urorectal septum, which precludes the appropriate separation of the embryonic cloaca into the anorectal and urogenital structures. An imperforate anus is the most common form of all. In female patients, it is associated with rovestibular, rectofourchette, rectovaginal, and rectoperineal fistula instead of forming a normal anal opening [1, 2].

In developed countries, early diagnosis leads to corrective surgery and improved functional outcomes. However, in resource‐limited settings due to limited access to antenatal and neonatal care, ARMs are diagnosed and treated later in life. This implies that patients present late in life with long‐standing physical, social, and psychological burdens [3, 4].

The case is primarily unique as it documents the presentation of a 24‐year‐old adult patient with an imperforate anus and a rectoperineal fistula. Thus, making it the oldest case ever reported with such a congenital anomaly. This provides an insight into the management challenges of adult patients with imperforate anus.

## 2. Case Presentation

We present a 24‐year‐old female patient from the Port Loko district of Sierra Leone. She was found by a screening team at Aberdeen Women’s Centre while they were doing screening for urogenital fistulas. She presented with the complaint of inability to control stool and the absence of an anal opening since childhood.

This case is unique because it describes the presentation of an imperforate anus in a 24‐year‐old patient, representing one of the oldest reported cases of this congenital anomaly in the literature, to the best of our knowledge.

She experienced stool incontinence and recurrent urinary tract infections. She was socially stigmatized and considered an outcast and cursed by the community. She had repeatedly stopped going to school because of the stigma of having stool incontinence. She did not seek any marriage because of the condition.

On physical examination, she had a normal general appearance and normal vital signs. The abdomen was flat, pelvic examinations revealed an imperforate anus with a visible raphe, and a rectoperineal fistula; the vagina appeared normal with a normal‐appearing cervix. The sacrum was palpable. She had no problem with gait. She had no cardiac or musculoskeletal abnormalities. Although preoperative imaging is commonly part of the workup for ARMs, it was not performed in this patient because the sacrum was palpably normal and there were no musculoskeletal abnormalities; in selected low‐ARM cases with a visible fistula, cross‐table lateral radiography may be omitted [5] (Figure 1).

**Figure 1:**
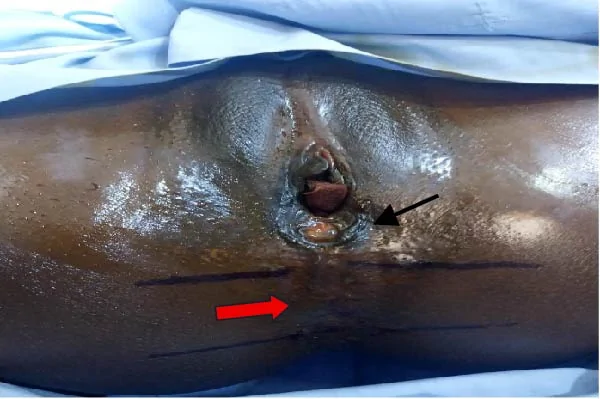
Imperforate anus with a rectoperineal fistula (black arrow, rectoperineal fistula; red arrow, center of the sphincter; blue dye margins of the sphincter).

Upon ultrasound examinations, both kidneys appeared normal, and a normal uterus and adnexa were identified. The full blood count was normal, with normal serological tests.

The patient was provided with counseling by the psychosocial workers. After written and informed consent was taken. The procedure was done under general anesthesia. Perineum‐sparing, anal transposition, and posterior sagittal anorectoplasty were done. The extent of the sphincter was delineated using a muscle stimulator and subsequently marked with a blue dye. The incision was started within the sphincteric region, and dissection was carried out until the rectum was identified. The rectum was mobilized for an adequate length posteriorly and laterally. The levator muscle complex, including the puborectalis muscle, was identified. Deep dissection was made posteriorly to locate and dissect the rectum. Then, a circumferential incision was made around the fistula, sparing the skin, and then dissected to join it with dissections posteriorly. Then, the fistula was delivered through the transsphincteric incision. Following the pull‐through of the dissected rectum/fistula, the posterior muscle complex was reapproximated deliberately to provide muscular coverage and support around the rectum. Then, anastomosis was made with the anal skin. The skin from the fistula was left intact, serving as a dentate line (Figures 2–6). On the same day, a loop sigmoid colostomy was done. The total estimated blood was less than 50 mL. Compared with neonatal anorectoplasty, this adult anorectoplasty required a deeper dissection to identify the rectum. The tissues were more vascular, and the intraoperative blood loss was greater. However, the sphincter complex was readily identifiable, which facilitated the maintenance of a symmetric dissection plane. Separation of the rectal tissue from the vagina around the fistula was particularly challenging because of the increased scar tissue.

**Figure 2:**
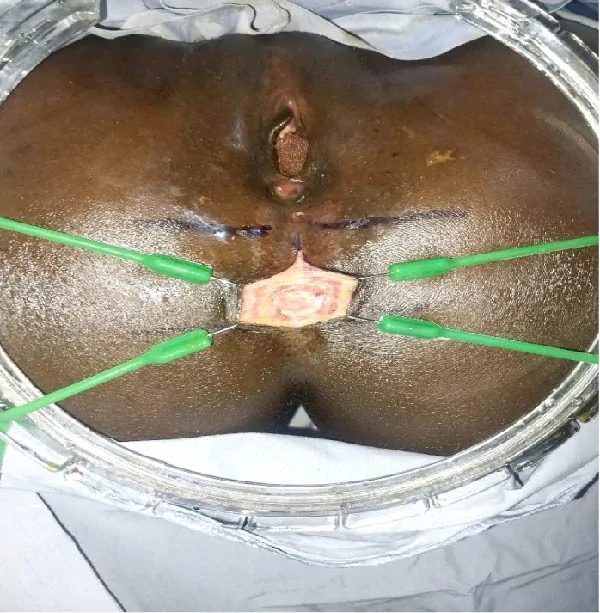
Intersphincteric incision.

**Figure 3:**
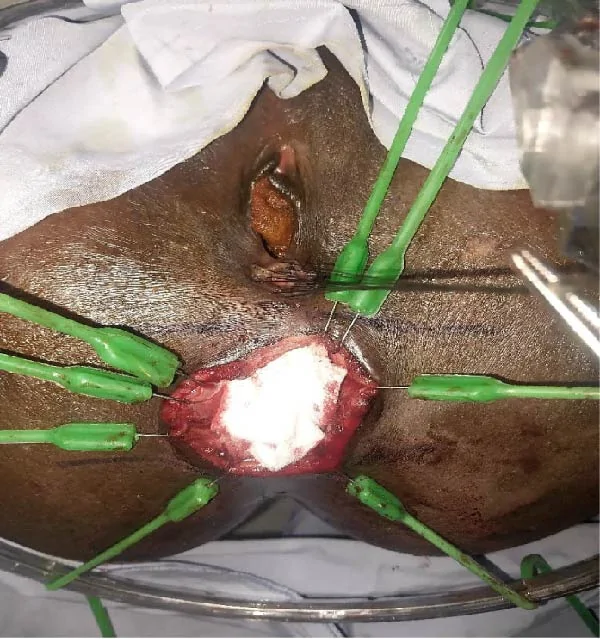
An incision is made until the rectum is reached, and a circumferential incision is made over the fistula to be made.

**Figure 4:**
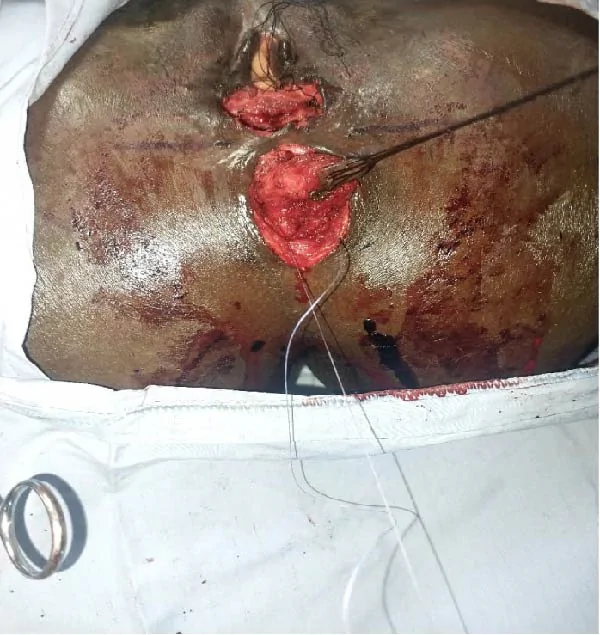
The fistula being transposed.

**Figure 5:**
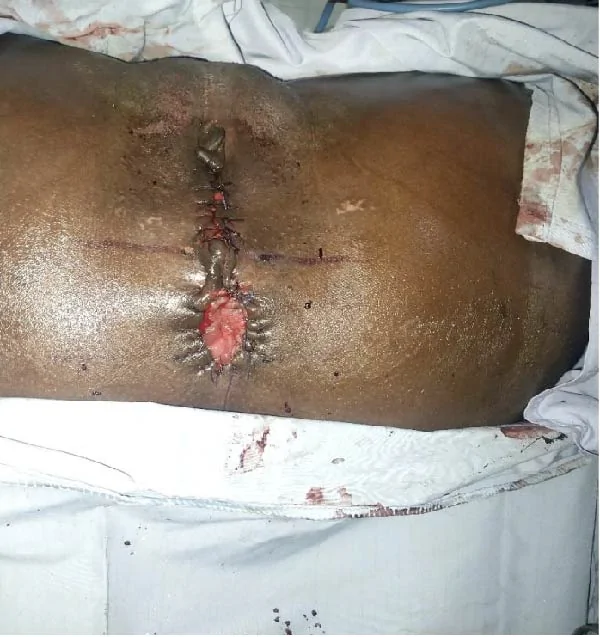
Anorectoplasty completed.

**Figure 6:**
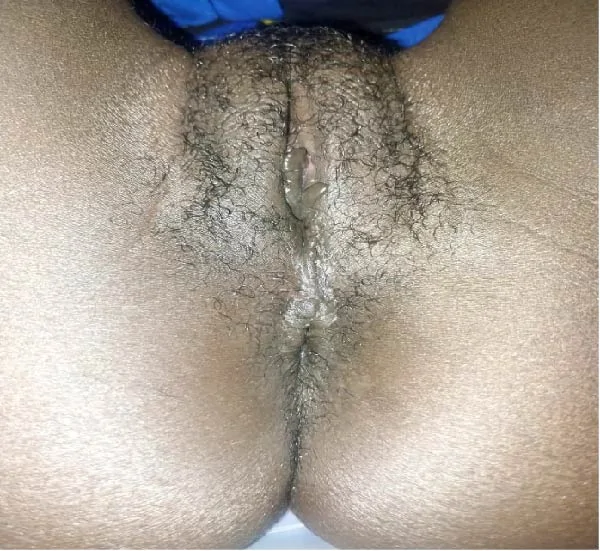
Nine months postoperative follow‐up of the anorectoplasty wound.

Once per week, anal dilatation was performed for 1 month. The loop colostomy was reversed 1 month after surgery. A distal loopogram was not performed. The patient was continent at discharge and was given lactulose 10 mL daily for 1 week. The patient presented for follow‐up at 3, 6, and 9 months. She was continent for stool and gas, could differentiate between gas and stool, and she had no episode of stooling. The wound healed well. Her social reintegration into the community was smooth, as she was trained in tailoring during her stay at Aberdeen Women’s Centre. She started a business in tailoring (Figures 2–6).

## 3. Discussion

Most of the ARMs are diagnosed during birth in developed countries as access to diagnosis and care is available. In low‐resource settings where home delivery is rampant, diagnosis is usually delayed, and patients often go undiagnosed until adulthood. Even if a family recognizes the abnormality, it usually accepts it as a curse or fate. Those who seek care will not obtain it as access to care is severely limited. Thus, patients with the same condition will bear the burden of physical, social, and psychological trauma for the remainder of their lives [6, 7]. This prolonged delay not only highlights a stark contrast in early childhood medical intervention but also amplifies the clinical intrigue behind selecting and adapting surgical techniques for adults who have lived with such conditions for years.

There are different ways of classifying ARMs. Modern classifications such as Krickenbeck describe the anatomy more precisely. While historically, Wingspread used classification such as high, intermediate, and low types. The present case would have been classified as low if the latter classification system was used [8–10].

Posterior sagittal anorectoplasty was first described by Alberto Pena in 1980 and revolutionized the management of ARM by directly accessing the malformations and surrounding structures [11, 12]. This has led to improved surgical outcomes, leading to improved continence and sexual function [13]. This approach requires extensive dissection until the level of the sacrum. Due to the extent of dissection and involvement of the perineal skin wound, the technique remains the major postoperative challenge [14, 15]. Furthermore, postprocedure frequent dilatation is quite distressing to the patient [16]. In the present case, the perineum was spared, and the fistula was transposed to the anus. The dissection was less extensive than that of Penas; thus, no perineal dehiscence was encountered. Unlike Peña’s original protocol, which recommends frequent postoperative dilatation, dilatation in this case was performed every week, reducing patient discomfort [17, 18].

However, Badillo et al. [19] reported a modified technique known as the perineum‐sparing posterior sagittal anorectoplasty. This approach avoids the incision of the perineal body by creating a new anal opening from the rectal wall and dropping the fistula without the need to transpose. Their findings suggest that this technique reduces the need for routine postoperative dilatation and reduces the risk of perineal body dehiscence [19, 20].

In the present case, the fistulous opening was transposed to the expected anal site rather than excised and replaced by a newly created neo‐anus. Similar transposition techniques for anterior ectopic anus have been described with preservation of the mucocutaneous junction as well as part of the local innervation and blood supply. This preserved mucocutaneous interface may represent a transition zone analogous to the dentate line, although its functional significance remains uncertain [21]. Literature on surgical ARM management for adult patients is limited [3, 6, 22]. In the present case, we followed most principles of PSARP, except for the need for deeper dissection due to anatomical changes.

Although this patient did not have a fecaloma, dissection in adult patients with delayed ARM may be more challenging when a fecaloma is present or when the distal rectum is markedly distended. Chronic fecal loading and distal fecal impaction have been associated with the presence of megarectum or megarectosigmoid, particularly in patients who have had a long interval between colostomy formation and definitive repair [23, 24]. Prior abdominal or colonic surgery can further complicate definitive anorectoplasty because adhesions, altered bowel length, and previous mobilization or resection may limit the available rectum or colon for pull‐through. In such cases, preservation of an adequate vascular pedicle is essential as the superior rectal artery is the principal blood supply to the rectum, with additional contribution from the middle and inferior rectal arteries to the distal rectum and anal canal [25]. Therefore, extensive dissection or previous surgery that compromises the rectal blood supply may make mobilization challenging.

Due to limited access to care, patients with ARM present late in low‐resource settings after facing psychosocial stigma [4]. Literature on how to approach adult patients with ARM surgically and psychosocially is limited [26]. This article presented a successful perineal‐skin‐sparing anal transposition posterior sagittal anorectoplasty as an approach to a 24‐year‐old patient. Thus, perineal‐sparing approaches should be utilized for adult ARM patients.

## Author Contributions

Awol Yemane Legesse contributed to conceptualization, investigation, formal analysis, writing – original draft, surgical management. Sia Nyandemo contributed to investigation, surgical management, writing – review and editing. Hale Teka and Mekonen Hafte contributed to writing – review and editing. Sherif Emil contributed to supervision, writing – review and editing, and provided specialized training and mentorship to Awol Yemane Legesse in pediatric anorectal malformation (ARM) surgery.

## Funding

No funding was solicited for the publication of this article.

## Consent

The patient provided written informed consent for publication.

## Conflicts of Interest

The authors declare no conflicts of interest.

## Data Availability

All relevant data are presented within the manuscript.
